# Embryo-lethal phenotypes in early *abp1* mutants are due to disruption of the neighboring *BSM* gene

**DOI:** 10.12688/f1000research.7143.1

**Published:** 2015-10-23

**Authors:** Jaroslav Michalko, Marta Dravecká, Tobias Bollenbach, Jiří Friml

**Affiliations:** 1 Institute of Science and Technology Austria (IST Austria), Klosterneuburg, 3400, Austria; 2 Institute of Plant Genetics and Biotechnology, Slovak Academy of Sciences, Nitra, 95007, Slovakia

**Keywords:** Auxin-binding protein 1, ABP1, BELAYA SMERT, BSM, embryo-lethality, allelism, short read DNA mapping

## Abstract

The Auxin Binding Protein1 (ABP1) has been identified based on its ability to bind auxin with high affinity and studied for a long time as a prime candidate for the extracellular auxin receptor responsible for mediating in particular the fast non-transcriptional auxin responses. However, the contradiction between the embryo-lethal phenotypes of the originally described *Arabidopsis* T-DNA insertional knock-out alleles ( *abp1-1* and *abp1-1s*) and the wild type-like phenotypes of other recently described loss-of-function alleles ( *abp1-c1* and *abp1-TD1*) questions the biological importance of ABP1 and relevance of the previous genetic studies. Here we show that there is no hidden copy of the *ABP1* gene in the *Arabidopsis *genome but the embryo-lethal phenotypes of *abp1-1* and *abp1-1s* alleles are very similar to the knock-out phenotypes of the neighboring gene, *BELAYA SMERT* ( *BSM*). Furthermore, the allelic complementation test between *bsm* and *abp1* alleles shows that the embryo-lethality in the *abp1-1* and *abp1-1s* alleles is caused by the off-target disruption of the *BSM* locus by the T-DNA insertions. This clarifies the controversy of different phenotypes among published *abp1* knock-out alleles and asks for reflections on the developmental role of ABP1.

## Introduction

The plant hormone auxin plays a central role in plant growth and development. Sensing and interpreting the fluctuating cellular auxin levels is crucial for mediating the corresponding physiological and developmental responses ( Enders & Strader, 2015; Grunewald & Friml, 2010). Currently, two main auxin receptor/co-receptor systems are known and have been proposed to activate a range of cellular responses; among them the Auxin Binding Protein 1 (ABP1) has been considered as a prime candidate for the extracellular auxin receptor ( Grones & Friml, 2015).

The first notion of ABP1 was based on the auxin-binding activity at the plant cell surface and in the endoplasmic reticulum in crude membrane preparations of etiolated coleoptiles ( Hertel *et al.*, 1972). This binding activity was characterized over the next decade by detailed biochemical studies ( Batt *et al.*, 1976; Ray *et al.*, 1977). ABP1 was firstly purified from maize coleoptile cells ( Lobler & Klambt, 1985) as a soluble 22-kDa large glycoprotein and later its crystal structure was elucidated ( Woo *et al.*, 2002).

The biological function and importance of ABP1 has been investigated extensively. Early studies have demonstrated that APB1 is involved in the rapid regulation of the membrane potential and ion fluxes at the plasma membrane and that it mediates the auxin-induced cell swelling, cell elongation, and cell division ( Braun *et al.*, 2008; Steffens *et al.*, 2001; Tromas *et al.*, 2009; Yamagami *et al.*, 2004). In the presence of auxin, ABP1 activates the H ^+^ pump ATPase that acidifies the extracellular space, presumably triggering cell wall loosening ( Cosgrove, 2000).

With the advent of the genomic era and *Arabidopsis thaliana* as a model system, genetic tools have been adopted to facilitate the studies of the ABP1 developmental roles. Using a reverse genetic approach, two independent *Arabidopsis thaliana* knock-out alleles of *ABP1* gene were identified, *abp1-1* and *abp1-1s* ( Chen *et al.*, 2001; Tzafrir *et al.*, 2004) and both were reported to be allelic and embryo-lethal, arresting the embryo development at the globular stage. This defined ABP1 as an essential protein required from early embryonic development with functions in cell division and elongation ( Chen *et al.*, 2001). However, embryo-lethal phenotype of *abp1* knock-out mutants has hampered its further functional characterization. This was rectified by the generation of transgenic lines conditionally downregulating ABP1 levels by ethanol-inducible immuno-modulation or antisense approaches that, despite entirely different technologies used, show the same phenotypes confirming the role of ABP1 in cell division and cell expansion ( Braun *et al.*, 2008; David *et al.*, 2007).

Both downregulation lines along with the weak *abp1-5* point mutation allele showed defects in clathrin-mediated endocytosis of PIN auxin export proteins ( Dhonukshe *et al.*, 2007; Petrášek *et al.*, 2006) and their inhibition by auxin ( Paciorek *et al.*, 2005). In contrast, the ABP1 gain-of-function mutation has an opposite effect, promoting PIN internalization in tobacco cultured cells and stable *Arabidopsis* lines ( Robert *et al.*, 2010). Opposite effects of *ABP1* loss- and gain-of-function lines were observed also for auxin effects on arrangements of microtubules ( Xu *et al.*, 2014) and for controlling morphogenesis and shape of leaf epidermal pavement cells ( Nagawa *et al.*, 2012; Xu *et al.*, 2010). Furthermore, the importance of auxin binding to ABP1 for gain-of-function phenotypes has been demonstrated by analysis of ABP1 variants with introduced mutations in the auxin binding pocket ( Grones *et al.*, 2015). Thus various types of loss- and gain-of-function strategies show an internally consistent picture of ABP1 signaling being involved in a range of physiological and cellular processes.

An important breakthrough came with the finding that the auxin-bound ABP1 docks on the extracellular domain of the Transmembrane Kinase 1 (TMK1) ( Dai *et al.*, 2013; Xu *et al.*, 2014) which added the missing piece to the puzzle of how the auxin signal is transmitted from the cell surface to the cytosol and further confirmed involvement of the ABP1/TMK1 pathway in auxin-mediated development, particularly in pavement cell morphology ( Grones & Friml, 2015).

Surprisingly enough, shortly after these studies had been published, Gao *et al.* (2015) described two independent, full loss-of-function *abp1* mutants of *A. thaliana* ( *abp1-c1* and *abp1-TD1*) that show no apparent developmental defects under normal growth conditions. This directly contradicts the embryo-lethal phenotypes of the originally described *abp1-1* and *abp1-1s* lines ( Chen *et al.*, 2001; www.seedgenes.org/SeedGeneProfile?geneSymbol=ABP+1) and also questioned the relevance of the aforementioned studies.

The explanation of these contradictory results is therefore of crucial importance to understand the biological role of ABP1. In this study we aim to clarify the discrepancy between the dramatic embryolethal phenotype of the originally described *abp1* knock-out mutants and the newly identified loss-of-function alleles.

## Material and methods

### Plant material and growth conditions

*Arabidopsis thaliana* mutants used in this study were: *abp1-1* ( Chen *et al.*, 2001), *abp1-1s* (NASC accession N16148), *abp1-c1*, *abp1-TD1* ( Gao *et al.*, 2015), *bsm1-1* ( Babiychuk *et al.*, 1997). *A. thaliana* Col-0 wild type seeds were obtained from The Nottingham Arabidopsis Stock Centre (NASC, http://arabidopsis.info). The seeds were vernalized for 3 days in the dark at 4°C. Plants were grown under long-day conditions (16-h light/8-h dark cycles) at 22°C in soil (Potgrond P) : perlit 4 : 1 substrate and watered regularly with tap water.

For selection of *abp1-1*, *abp1-1s* and *bsm1-1* heterozygous plants, seeds were surface-sterilized with chlorine vapor and plated on 1/2 MS agar medium (pH 5.9) containing 1% (w/v) sucrose and 25 µg/mL kanamycin according to Harrison *et al.* (2006).

### Coverage at the ABP1 locus

**Figure 1.**
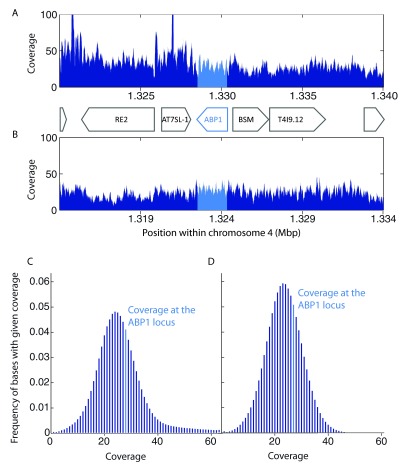
*In silico* mapping showed normal short read coverage at the *ABP1* locus. To test the hypothesis of hidden *ABP1* duplicates in the genome of *Arabidopsis thaliana,* two short reads libraries from *A. thaliana* re-sequencing projects (NCBI accession numbers SRX759525 and SRX703650) were separately aligned to the reference *Arabidopsis* genome (TAIR10) using the BOWTIE 2 suit using different mapping parameter sets (see the Methods section). ( **A, B**) The coverage of the 20 kbp consensus sequence within the chromosome 4 that surrounds *ABP1* is shown for the two libraries: SRX759525 ( **A**) and SRX703650 ( **B**). ( **C, D**) The overall distribution of the base coverage within the chromosome 4 is shown below. The median coverage at the *ABP1* locus is highlighted in light blue and is well within the expected coverage values for both SRX759525 ( **C**) and SRX703650 ( **D**).

Data from two publicly available short read libraries (NCBI Short Reads Archive accessions SRX759525 and SRX703650) from *A. thaliana* re-sequencing experiments were downloaded and mapped to the deposited reference genome TAIR10 (accessions NC_003070, NC_003071, NC_003074, NC_003075, NC_003076) using the Bowtie 2 plugin within the Geneious software, version 7.1.7 ( http://www.geneious.com). For the first analyzed short read library (accession SRX759525), mapping parameters were set to a local alignment (“--very-fast-local”) with multi-mapping allowed (value k = 10). For the second analyzed short read library (accession SRX703650), mapping parameters were set to end-to-end alignment and the best-match mapping option (k = 1) was used. In both cases the seed length (L) was set to 22 and number of mismatches (N) to 1. To maximize the mapping coverage short reads were mapped single-end. For all the remaining parameters the default Bowtie 2 values were used. For both alignments, the median coverage at the *ABP1* locus (bases 1 319 656 - 1 321 477 of NC_003075) was compared to the surrounding 20 Kbp region ( Figure 1A and 1B) and to the whole chromosome 4 ( Figure 1C and Dataset 2 for SRX759525; Figure 1D and Dataset 3 for SRX703650). Mapping coverage is defined as the number of reads mapped to a given base. Coverage data from the Geneious software were exported and visualized using Matlab (v. R2011b).

### Genotyping mutants

The T-DNA insertional mutants were genotyped by using a PCR-based method ( Alonso *et al.*, 2003). Amplification of PCR products was made using Phire Plant Direct PCR Kit (obtained from Thermo Scientific by Finnzymes, Espoo, Finland) following manufacturer’s instructions for the dilution protocol and using Bio-Rad T100 Thermal Cycler. The PCR conditions were as follows: initial denaturation for 5 min. at 98°C and subsequent 40 cycles: denaturation for 5 s at 98°C, annealing for 10 s at the respective annealing temperature for each primer set (calculated with the Thermo Scientific Tm calculator; available at www.thermofisher.com), elongation for 30 s at 72°C and final elongation for 1 min. at 72°C.

Genotyping primers were as follows: cdsBSM_F and nptII_R for the *bsm* mutation, ABP1_3UTR_FOR and WiscDsLoc_REV for the *abp1-1* mutation, WiscDsLoc_REV and qBSM_R2 for the *abp1-1s* mutation. Genotyping of *abp1-c1* and *abp1-TD1* mutants was described previously ( Gao *et al.*, 2015). Sequences of all primers used in this study are listed in Table 1. Purified PCR products from plants positive for the presence of the T-DNA insertion were sequenced using a commercial service (LGC genomics, www.lgcgenomics.com). Primers used for sequencing were the same as for PCR. Sequence reads were aligned against *A. thaliana* genome (TAIR 10) using the BLAST tool ( http://blast.ncbi.nlm.nih.gov/Blast.cgi). The position of the mutation was identified at the border of the sequence part aligned to *ABP1* locus and the sequence part aligned to the transformation vector. Position of individual mutations was mapped to the sequence of *ABP1/BSM* locus acquired from the TAIR database ( https://www.arabidopsis.org), visualized with the SnapGene Viewer software version 2.8.2 ( http://www.snapgene.com/products/snapgene_viewer) and edited in MS Power Point 2010.

**Table 1.** Primers used in this study.

Name	Sequence (5’-3’)	Target
Genotyping primers		
pSKTAIL-L3	ATACGACGGATCGTAATTTGTCG	
ABP1-U409F	CCTCATCACACAACAAAGTCACTC	
ABP1-586R	GGAGCCAGCAACAGTCATGTG	
cBSM_F	AAAAAACCTTACCCGCTCCTCTAA	
nptII_R	AGCCAACGCTATGTCCTGAT	
ABP1 3-UTR_FOR	GTATCTACGTAGTGTCACAAAACCTCAAC	
WiscDsLoc_REV	TCCCAACAGTTGCGCACCTGAATG	
qBSM_R2	CCCAGGCTTTGTGAAGCCATTAC	
		
Primers for qRT-PCR		
qBSM_F2	TTTCCTCAGCTCCGGTAAAGAATG	*BSM* gene (At4g02990)
qBSM_R2	CCCAGGCTTTGTGAAGCCATTAC	
A2E	TTGCCAATCGTGAGGAATATTAG	*ABP1* gene (At4g02980)
ABP1-586R	GGAGCCAGCAACAGTCATGTG	
TUB-2_F3	TAACAACTGGGCCAAGGGACAC	*TUB2* (At5g62690)
TUB-2_R3	ACAAACCTGGAACCCTTGGAGAC	

### Quantitative RT-PCR

For the RNA extraction approximately twenty 8 day-old seedlings were frozen in liquid nitrogen. Total RNA was extracted using the TRIzol reagent (Invitrogen, Carlsbad, CA, USA) and purified using RNeasy Mini Kit (Qiagen) according to manufacturer’s instructions. For digestion of genomic DNA the following modification was incorporated into the protocol: in step 7, first 350 µL of RW1 buffer was added to the RNeasy spin column and centrifuged (8000 rpm, room temperature, 15 s). Subsequently, 40 µL of the RNase-free recombinant DNase I incubation mix (mixture of 5 µL DNase I and 35 µL buffer RDD) (Roche) was added to the column and incubated for 15 min at room temperature. Next, another 350 µL of RW1 buffer was added to the column and centrifuged again (8000 rpm, room temperature, 15 s). Two µg of purified DNase I pre-treated total RNA was used for a reverse transcription reaction using the iScript cDNA Synthesis Kit (BioRad, Hercules, CA, USA). Primers used for the quantitative RT-PCR were designed using QuantPrime ( http://www.quantprime.de). For amplification of the *BSM* cDNA fragment (88 bp in length) primers qBSM_F2 and qBSM_R2 were used. The *ABP1* cDNA fragment (84 bp in length) was amplified with A2E and ABP1-586R primers. *Arabidopsis tubulin beta chain 2* ( *TUB2*, At5g62690) was used as a reference gene and amplified with TUB-2_F3 and TUB-2_R3 primer set ( Table 1, Dataset 1). All primers were synthesized by commercial service ( https://www.eurofinsgenomics.eu/). qRT-PCR was performed using the LightCycler 480 SYBR Green I Master chemistry (Roche) in a LightCycler 480 II thermal cycler (Ser. no. 5659, Roche) according to manufacturer’s instructions. A 1:10 cDNA dilution was used as a template to prepare 5 µL reaction mixture (final volume). The cycling conditions were as follows: pre-incubation for 10 min. at 95°C and subsequent 45 cycles: denaturation for 10 s at 95°C, annealing for 15 s at 60°C, elongation for 15 s at 72°C followed by high resolution melting analysis. Gene expression was calculated with the 2-ΔΔCT method ( Livak & Schmittgen, 2001). Individual experiments were made in a technical triplicate.

### Analysis of embryo defects

To compare the stage of embryo arrest, seeds from siliques of the heterozygous *abp1-1* and *bsm* plants in the 7 ^th^ to 8 ^th^ developmental stage were used. For evaluation of embryo development, seeds from the siliques in the 3 ^rd^ to 5 ^th^ developmental stage were used. Siliques were dissected using a sharp needle and seeds were extracted and transferred onto microscopic slides into the drop of the Hoyer’s solution ( http://www.seedgenes.org/Tutorial.html) and cleared for 1 h similarly as described ( Friml *et al.*, 2003). The embryos were analyzed under 20 × magnification using a digital camera system of the Olympus BX53 microscope by Normanski optics (Differential Interference Contrast (DIC) light microscopy). Images were processed in the ImageJ software, v. 1.48 ( http://imagej.nih.gov/ij/) and mounted in Adobe Illustrator CS5.1.

### Allelic test

Approximately 4 week-old plants were used for crossing. Flowers of recipient plants were emasculated 2 days before pollination. Crosses were generated by manual pollination. Green siliques were dissected 8 days after pollination and number of white and green seeds was counted and their ratio calculated.

## Results

### No hidden copy of the *ABP1* gene can be found in the genome of *A. thaliana*

One of the possibilities explaining at least some of the recent ABP1 controversies, in particular the strong phenotypes of the conditional lines versus no apparent phenotypes of the *abp1-c1* and *abp1-TD1* alleles, is the presence of a second, non-annotated *ABP1* gene copy in the genome of *A. thaliana* that is functionally redundant and not disrupted in the *abp1-c1* and *abp1-TD1* alleles. Highly similar copies of the *ABP1* gene are present in the annotated genomes of maize ( *Zea mays*), rice ( *Oryza sativa*) or poplar ( *Populus trichocarpa*) ( http://phytozome.jgi.doe.gov). During genome assembly, highly similar sequences such as transposons or recently duplicated genes could collapse into one copy resulting in no annotation of the duplicated copy (Stephane Rombauts, personal communication, 30 ^th^ January 2015). A non-annotated second copy of the *ABP1* gene is also present in the sequenced genome of the moss *Physcomitrella patens* ( http://phytozome.jgi.doe.gov) raising the possibility that a similar situation could take a place in the *A. thaliana* genome.

We hypothesized that in case of a second non-annotated copy of *ABP1* in the *A. thaliana* genome, the short reads coverage at the *ABP1* locus would be doubled compared to the neighboring DNA regions and to the coverage of most other loci on the chromosome. To address this question we mapped short reads from two publicly available short read libraries from *A. thaliana* re-sequencing projects to the annotated *Arabidopsis* reference genome (TAIR 10) and analyzed the coverage at the *ABP1* locus.

As depicted ( Figure 1), the number of aligned short reads at the *ABP1* locus is comparable with the short read coverage of the neighboring sequences and is well within the range of most common coverage values for loci on chromosome 4 indicating that no hidden copy of *ABP1* is present in the *A. thaliana* genome.

### *ABP1* is tightly linked with a closely-located gene encoding for plastid-localized mTERF protein BSM important for embryogenesis

**Figure 2.**
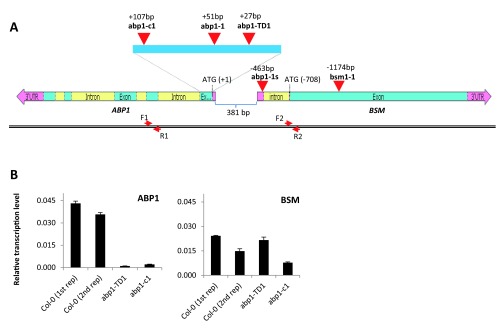
*ABP1* is tightly linked with its neighboring gene *BSM*. ( **A**) Map of the *ABP1/BSM* locus showing the mapped position of existing loss-of-function mutant alleles (red arrowheads) relative to the *ABP1* translation start. Note that the T-DNA insertion in *abp1-1s* allele is located in the 5’-UTR region of the *BSM* gene. Positions of the forward primers ABP1-586R (F1) and qBSM_F2 (F2) and reverse primers A2E (R1) and qBSM_R2 (R2) used for the qRT-PCR are indicated by red arrows. ( **B**) Quantitative real-time PCR of the *ABP1* (left graph) and *BSM* (right graph) shows relative transcript levels in wild-type Col-0, *abp1-c1* and *abp1-TD1* seedlings. The reference gene was *TUB2* (At5g62690). The transcript levels were calculated using 2-ΔΔCT method. The data represent average relative quantity values of three technical replicates, and the bars denote standard errors. For wild-type Col-0 plants data from 2 biological replicates are shown.

Another possibility to explain phenotypic discrepancies between different *abp1* mutant alleles is a disruption of another gene in addition to *ABP1* that would be responsible for the strong phenotypes in the *abp1-1* and *abp1-1s* alleles. As it can be seen on the *ABP1* locus map ( Figure 2A), the *ABP1* gene is closely located to its neighboring gene *BSM* in a head-to head orientation with translational start codons and 5’-UTR regions of the two genes being just 708 bp and 381 bp respectively apart from each other. *BSM* encodes the mitochondrial transcription termination factor (mTERF)-like protein responsible for mitochondrial transcription termination in humans. In plants, mutants disrupting the *BSM* gene have been shown to be embryo-lethal ( Babiychuk *et al.*, 2011), therefore we suspected that the *BSM* gene is the most likely off-target of mutations in the *abp1-1* and *abp1-1s* alleles.

To look into this possibility closely, we mapped the relative position of individual mutations within the *ABP1/BSM* locus ( Figure 2A). Surprisingly, we found that the T-DNA insertion in *abp1-1s* mutant is in fact located in the 5’-UTR of the *BSM* gene. Because *ABP1* and *BSM* share a common promoter region, we hypothesized that the T-DNA insertion in *abp1-1* could also disrupt the expression of the *BSM* gene thus causing the described embryonic lethality. Notably, the T-DNA insertion in the wild type-like allele *abp1-TD1* is located just 24 bp apart from the *abp1-1* T-DNA insertion and is even closer to the *BSM* gene. We speculated that the dramatic phenotypic difference between both closely positioned T-DNA-based insertions might be caused by the presence of multiplied 35S enhancers within the T-DNA construct in the *abp1-TD1*, because this allele was recovered from the activation tagging *Arabidopsis* mutant collection (SASKATOON) and harbors multiplied enhancers near the right T-DNA border on the inserted T-DNA element ( Figure 2B).

To see whether the expression of the *BSM* gene in homozygous *abp1-TD1* plants was affected by the T-DNA insertion, we performed a qRT-PCR analysis. Surprisingly, the *BSM* transcript levels in *abp1-TD1* were not substantially affected by the T-DNA insertion and were comparable to wild type Col-0 plants ( Figure 2B). On the other hand, the expression of the *ABP1* gene was abolished in both *abp1-c1* and *abp1-TD1* homozygous plants as reported previously ( Gao *et al.*, 2015). For *abp1-1*, *abp1-1s* or *bsm* mutants it was only possible to evaluate the *BSM* expression in the heterozygous plants (due to the lethality of homozygous mutants) and this showed no substantial differences compared to wild type plants ( Dataset 1).

Therefore, it is possible that while in the *abp1-TD1* allele the expression of the *BSM* gene is rescued by its ectopic expression driven by 35S enhancers within the T-DNA, in the *abp1-1* allele, where no such a mechanism exists, the expression of both genes may be disrupted. In addition, the *abp1-c1* null allele only contains a small 5 bp deletion at the end of the first exon of *ABP1* which is less likely to have any effect on the *BSM* gene compared to a few kB long T-DNA insertions in other alleles. Thus it is plausible that in *abp1-1* and *abp1-1s*, both *ABP1* and *BSM* genes are disrupted.

### *abp1* and *bsm* knock-out mutants show similar defects in embryo development

To compare the embryo-lethal phenotypes of *abp1-1* and *abp1-1s* alleles with the embryo-lethal phenotype of the *bsm1-1* T-DNA insertional knock-out allele, we analyzed the embryo development in siliques of *abp1-1*, *abp1-1s* and *bsm1-1* heterozygous plants along with homozygous new *abp1* knock-out alleles.

The inactivation of *BSM* gene by T-DNA insertion has been shown to cause an embryo arrest at the late globular stage of development ( Babiychuk *et al.*, 2011). In the *abp1-1* mutants the embryo arrest has been reported at the early globular 32-cell stage in 25% of immature seeds ( Chen *et al.*, 2001). In both cases the homozygous mutant embryos never reach the heart-shape stage, indicating prevention in vertical elongation and subsequent anticlinal placement of division planes in the lower tier of cells. Other characteristic features reported in *abp1-1* embryos have been connected with failure in degeneration of the suspensor structure and ectopic anticlinal divisions of basal cells resulting in longer suspensors at the later stages ( Chen *et al.*, 2001).

**Figure 3.**
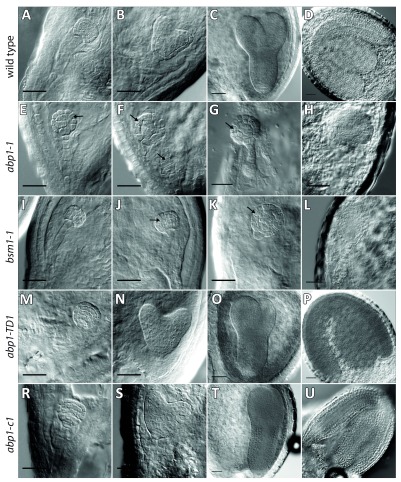
*abp1-1* and *bsm* mutants show similar defects during embryo development. Seeds from the siliques of *abp1-1+/-* ( **A-H**), *bsm+/-* ( **I**– **L**), *abp1-TD1 -/-* ( **M**– **P**), and *abp1-c1* -/- ( **R**– **U**) plants at different developmental stages were isolated and cleared in Hoyer’s solution and screened for defects in embryo development using Normanski optics. Wild type-looking embryos in the seeds of *abp1-1* mutant ( **A**– **D**) showed normal developmental progression: the late globular stage ( **A**), early heart ( **B**), torpedo ( **C**) as well as the mature embryo stage ( **D**). At the globular stage we started to observe differences in embryo development between different mutants earliest manifested by periclinal divisions in protodermal cells (arrows) ( **A, E, I, M**). When most of the wild-type embryos entered the early heart stage ( **B**) some *abp1-1* embryos, from the same silique as ( **B**) showed abnormal cell divisions ( **F**), also visible in *bsm* ( **J**) (arrows) but not in the *abp1-TD1* ( **N**) or *abp1-c1* mutant ( **S**). At the later stages of embryo development, when wild-type embryos in *abp1-1* ( **C, D**) as well as embryos in *abp1-TD1* ( **O, P**) and *abp1-c1* ( **T, U**) reached the torpedo ( **C, O, T**) and mature stage ( **D, P, U**), the mutant *abp1-1* ( **G, H**) and *bsm* ( **K, L**) embryos were still arrested at the late globular stage. While the *bsm* mutant embryos show signs of apical basal polarity, *abp1-1* embryos formed ball-shaped symmetric structure. Also, abnormal cell divisions in the suspensor cells were visible (arrows). Scale bars, 25 µm.

Inspection of embryos at different stages from the homozygous *abp1-c1* and *abp1-TD1* plants did not reveal any aberrations in the embryo development as compared to the wild type embryos ( Figure 3) consistent with the lack of obvious postembryonic phenotypes ( Gao *et al.*, 2015). Also, no white aborted seeds were found in the older siliques of these plants ( Supplementary Figure 1).

**Figure 4.**
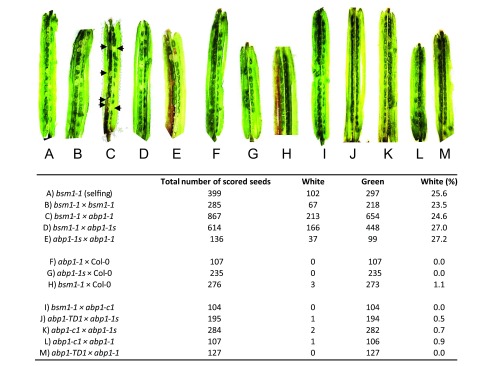
Allelic test between *A. thaliana bsm* and *abp1* knock-out mutants. The embryolethal phenotype was detected in siliques 8 days after pollination by the presence of white seeds (arrested embryo development). It was not possible to complement the embryo-lethal phenotype of *bsm* mutant ( **A, B**) with either *abp1-1* or *abp1-1s* mutant ( **C, D**). Similarly, *abp1-1* and *abp1-1s* mutants did not mutually complement ( **E**). As a control Col-0 plants were crossed into the *abp1-1* ( **F**), *abp1-1s* ( **G**) or *bsm* ( **H**) mutants showing complementation of the embryo-lethal phenotype. Genetic crosses of the recently identified *abp1* null mutants ( *abp1-c1*, *abp1-TD1*) with *bsm* ( **I**), *abp1-1s* ( **J, K**) or *abp1-1* ( **L, M**) lines resulted in complementation of the embryo-lethal phenotypes showing that in these lines the disruption of the *BSM* and not the *ABP1* gene is responsible for the embryo-lethal phenotype. Arrows indicate the position of white seeds in ( **C**). Number of scored seeds for each cross and the percentage of white seeds are listed in the table below. Note that in not complementing crosses, the segregation of embryo-lethal seeds follows the Mendelian genetic laws as expected.

Opening and inspection of the older siliques in *abp1-1*, *abp1-1s* and *bsm1-1* heterozygous plants did not reveal any visible differences between those alleles. In all three cases, the siliques carried approximately 25% of white aborted seeds (see Figure 4) containing developmentally arrested embryos ( Figure 3). Analysis of the younger embryonic stages did not reveal any significant differences between the analyzed alleles. The obvious embryo defects at the early globular stage were infrequent, and in both *abp1* and *bsm* mutants started to be more pronounced at the late globular stage manifested by the failure of directional cell elongation leading to ball-shaped embryos at later stages. Another characteristic feature for both mutants was a disrupted cell division pattern with frequent periclinal divisions of outer layer cells ( Figure 3B). At the later stages of embryo development, *bsm* mutant embryos showed some degree of apical-basal polarity by forming sometimes oval-shaped structures while *abp1-1* mutant embryos continue to divide non-directionally forming more symmetrically ball-shaped embryos. These differences could be explained either by different backgrounds of the two mutant alleles (C24 in the case of *bsm* and Wassilewskija in the case of *abp1-1*, ** respectively) or by simultaneous disruption of both *ABP1* and *BSM* genes in the *abp1-1* mutant that may produce stronger effect as compared to a single *BSM* loss-of-function mutation in *bsm1-1*. Despite these minor differences at the very late embryo stages, both mutations generated undistinguishable cell elongation and cell division pattern defects starting in both cases at the globular stage resulting in similar embryo-lethal phenotypes.

### Dysfunction of the *BSM* gene is responsible for embryonic lethality of the *abp1-1* and *abp1-1s* mutants

As the embryo-lethal phenotypes of *abp1* and *bsm* mutants are indistinguishable from each other, we tested whether these phenotypes might be due to the mutations affecting the same gene or they are independently embryolethal. To address this hypothesis we performed allelic crosses between the *bsm* and *abp1-1* as well as *abp1-1s* line and looked for the presence of white seeds carrying defective embryos within immature siliques 8 days after pollination of emasculated flowers. We assumed that if the mutations affect the same gene it will not be possible to complement the embryolethal phenotype of *abp1-1* or *abp1-1s* mutants with a functional *ABP1* allele from the *bsm* mutant which will result in the segregation of approximately 25% not developing (white) seeds in the F1 generation.

After dissection of siliques of *bsm1-1* × *abp1-1*, *bsm1-1* × *abp1-1s* or control crosses *bsm1-1* × *bsm1-1* and *abp1-1s* × *abp1-1*, we observed the presence of clearly distinguishable white seeds ( Figure 4A–E). The segregation ratio of 1:4 white:green seeds was observed (Table in Figure 4, Supplementary Table 1) which is in perfect agreement with the Mendelian genetic laws implying that *abp1-1* and *abp1-1s* mutants do not carry a functional *BSM* gene. On the other hand, when crossed with wild type (Col-0), *abp1-c1* or *abp1-TD1* mutants, more than 99% of seeds in the siliques were green, indicating that a functional copy of the *BSM* gene present in these lines was able to complement the embryolethal phenotype of *bsm1-1*, *abp1-1* and *abp1-1s* lines ( Figure 4F–M).

In summary, these observations show that the described embryolethal phenotype of the *abp1-1* and *abp1-1s* lines is caused by a disruption in the *BSM* gene.

Dataset 1 Quantitative RT-PCR data of the *BSM* and *ABP1* gene expression level in different mutant lines ( Michalko *et al.*, 2015a). Click here for additional data file. Copyright: © 2015 Michalko J et al. 2015 Data associated with the article are available under the terms of the Creative Commons Zero "No rights reserved" data waiver (CC0 1.0 Public domain dedication).

Dataset 2 The short read coverage of individual bases at their respective positions within the Chromosome 4 of the *Arabidopsis thaliana* genome based on the mapping of short reads from *Arabidopsis* re-sequencing project (NCBI number: SRX759525) to the reference *Arabidopsis* genome (version TAIR10) ( Michalko *et al.*, 2015b). Click here for additional data file. Copyright: © 2015 Michalko J et al. 2015 Data associated with the article are available under the terms of the Creative Commons Zero "No rights reserved" data waiver (CC0 1.0 Public domain dedication).

Dataset 3 The short read coverage of individual bases at their respective positions within the Chromosome 4 of the *Arabidopsis thaliana* genome based on the mapping of short reads from *Arabidopsis* re-sequencing project (NCBI number: SRX703650) to the reference *Arabidopsis* genome (version TAIR10) ( Michalko *et al.*, 2015c). Click here for additional data file. Copyright: © 2015 Michalko J et al. 2015 Data associated with the article are available under the terms of the Creative Commons Zero "No rights reserved" data waiver (CC0 1.0 Public domain dedication).

## Discussion

### Embryo-lethal phenotypes of early *abp1* knock-outs are caused by disruption of *BSM*

Since its discovery, the biological importance of the ABP1 protein as a plasma membrane auxin receptor has been a matter of debate, in part because of its predominant ER localization in plant cells where the conditions for auxin binding are unfavorable ( Habets & Offringa, 2015; Napier *et al.*, 2002). Nonetheless, after the two independently isolated *Arabidopsis abp1* loss-of-function alleles were reported to be embryolethal ( Chen *et al.*, 2001; www.seedgenes.org/SeedGeneProfile?geneSymbol=ABP+1), the crucial importance of this protein in cell division and elongation was accepted. This view was challenged by the isolation of two new knock-out alleles ( Gao *et al.*, 2015) that harbor mutations in a close vicinity to the previously published insertions and show no obvious phenotypes.

The phenotype of the originally reported *abp1-1* mutant was reported to be complemented by the *35S::ABP1* overexpression construct ( Chen *et al.*, 2001). However, despite multiple attempts it was not possible to repeat this complementation either with constructs for overexpression of the wild type *ABP1* copy or by the genomic fragment ( Grones *et al.*, 2015). Altogether, these findings implied that there exists another cause for the drastic phenotypes present in the *abp1-1* and *abp1-1s* mutants.

Here we show that the neighboring gene *BSM*, which has been shown previously to be crucial for embryogenesis in *Arabidopsis* ( Babiychuk *et al.*, 2011) is likely to be also disrupted by the T-DNA insertions in the original *abp1-1* and *abp1-1s* mutants, but not in the newly isolated loss-of-function lines *abp1-c1* and *abp1-TD1*. Furthermore, we demonstrate that the mutant embryos of *abp1-1* and *abp1-1s* as well as the loss-of-function *bsm1-1* allele showed similar embryo phenotypes and are arrested at the globular stage as it was shown in the original studies ( Babiychuk *et al.*, 2011; Chen *et al.*, 2001).

By allelic complementation experiments we showed that the embryolethal phenotype of the original *abp1-1* and *abp1-1s* alleles can be complemented by the functional copy of the *BSM* gene that is present in the new *abp1-c1* and *abp1-TD1* alleles, but not by the *bsm* loss-of-function or the *abp1-1* mutants themselves. Therefore, the originally described *abp1* mutants are, indeed, loss-of-function alleles of the *BSM* gene and at least *abp1-1* is a double loss-of-function mutant of *ABP1* and *BSM*. Thus, the embryo-lethal phenotype previously attributed to the *abp1* loss-of-function alleles is a result of the mutation in the neighboring *BSM* gene. For this reason, we also propose to re-annotate the *abp1-1* and *abp1-1s* alleles as *abp1-1*/ *bsm1-2* and *bsm1-3* (for this line it remains unclear if the insertion disrupts both *ABP1* and *BSM* expression), respectively.

### Functional importance of the ABP1 pathway

The *abp1* and *bsm* allelic test together with no embryonic defects in the true *abp1* knock-out lines makes it clear that no role has been identified for ABP1 in early embryogenesis. However, this clarification of *abp1* knock-out genotypes does not *per se* undermine all experimentation with *ABP1* genetic tools. The other *ABP1* genotypes comprise conditional and constitutive gain-of-function alleles, two types of conditional knock-downs, as well as a weak *abp1-5* point mutation ( Braun *et al.*, 2008; Covanova *et al.*, 2013; Grones *et al.*, 2015; Robert *et al.*, 2010; Tromas *et al.*, 2009; Xu *et al.*, 2010). In several analyzed cellular processes including auxin-dependent ROP-GTPase activation, auxin-regulated endocytosis, E3 ligase-mediated ubiquitination, or cortical microtubule reorientation ( Robert *et al.*, 2010; Sassi *et al.*, 2014; Tromas *et al.*, 2013; Xu *et al.*, 2010; Xu *et al.*, 2014) this genetic material produced fully internally consistent data sets. Furthermore, loss-of-function mutants in TMK receptor-like protein kinases, which interact with ABP1 in an auxin-inducible manner, show largely overlapping phenotypes with *abp1* mutants ( Xu *et al.*, 2014). Altogether, these data support the importance of the ABP1/TMK auxin perception and signaling mechanism in plant development and physiology. One possible explanation for the absence of obvious phenotype defects in true *abp1* knock-outs is a presence of another copy of the *ABP1* gene in the genome of *A. thaliana* that could escape the gene annotation during genome assembly and that could stay undetected by Southern blot analysis if the two copies were located close together. However, by *in silico* analysis of the *ABP1* locus coverage we excluded this possibility. Other possible ways to reconcile the absent phenotype defects in the knock-outs with observations from the other genetic material include a genetic compensation mechanism that can be triggered by independent deleterious loss-of-function mutations but not by the genetic knock-downs of the same genes as shown for example in zebrafish ( Rossi *et al.*, 2015). Such compensation machinery could involve previously largely overlooked ABP1-like proteins from the germin family that are not a close sequence homologs of ABP1 but share some common characteristic features and some of them have been identified by their binding to auxin (reviewed in Napier *et al.*, 2002).

Importantly, with the controversy of the embryonic phenotypes in different *abp1* mutant alleles clarified, we can move on and with the updated genetic toolbox clarify the role of ABP1 in auxin signaling and plant development. The high affinity binding of ABP1 to auxin, universal occurrence of the *ABP1* genes in the genomes from algae to higher plants and its highly conserved structure argue for its importance throughout the plant kingdom and promise further interesting discoveries.

## Data availability

The data referenced by this article are under copyright with the following copyright statement: Copyright: © 2015 Michalko J et al.

Data associated with the article are available under the terms of the Creative Commons Zero "No rights reserved" data waiver (CC0 1.0 Public domain dedication). 

*F1000Research*: Dataset 1. Dataset 1, 10.5256/f1000research.7143.d104552

*F1000Research*: Dataset 2. Dataset 2, 10.5256/f1000research.7143.d104553

*F1000Research*: Dataset 3. Dataset 3, 10.5256/f1000research.7143.d104554

## Supplementary materials

### Supplementary Table 1

Allelic test between *A. thaliana bsm* and *abp1* knock-out mutants. Complete table with number of scored seeds for all crosses made. The percentage of white seeds from the total number of scored seeds is shown in brackets for each tested combination. N/A = data not available,♀ = plant was used as a pollen recipient, ♂ = plant was used as a pollen donor.

### Supplementary Figure 1

Dissected immature siliques of *abp1-TD1* and *abp1-c1* mutants.
