# Supplementary material for: Embryo-lethal phenotypes in early abp1 mutants are due to disruption of the neighboring BSM gene
Source: F1000Res. 2015 Oct 23;4:1104. [Version 1] doi: 10.12688/f1000research.7143.1 (PMC4642851; doi:10.12688/f1000research.7143.1)
Supplement: Peer review history

# Peer review: Referee response for version 1

**DOI:** 10.5256/f1000research.7695.r10913

**Published:** 2015-11-02

**Authors:** Christian Luschnig

**Affiliations:** 1 Department of Applied Genetics and Cell Biology, University of Natural Resources and Life Sciences, Vienna, Austria

After publication of an article by Gao and colleagues earlier this year, several published results dealing with the role of *ABP1* in auxin signaling and plant development became a matter of doubt. This also concerns the embryo-lethal phenotypes that have been attributed to a loss of *ABP1* function in *abp1-1* and *abp1-1s* alleles - quite the opposite of the wild type appearance of the likely *abp1-1c* and *abp1-TD1* null alleles, described by Gao and colleagues. These contradicting results predict off-target effects that would cause phenotypes associated with *abp1-1* and *abp1-1s,* and the authors of this study attempted to identify such targets.

From the results presented in this manuscript it appears that functional disruption of the *BSM* gene, located proximal to *ABP1*, is responsible for *abp1-1*/ *abp1-1s* embryo-lethal phenotypes. This is indicated by genetic complementation analyses, demonstrating that neither *abp1-1* nor *abp1-1s* complement segregation of *bsm1-1* embryo-lethal phenotypes, when analyzing progeny of crosses. On the other hand, when introducing *bsm1-1* into *abp1-c1* and *abp1-TD1*, the authors observed complementation of embryo-lethal phenotypes. This provides genetic evidence for a scenario in which phenotypes associated with *abp1-1 *and *abp1-1s *result from disruption of the *BSM1* locus.

This is an important and timely report, summarizing experiments that certainly will contribute to our understanding of *ABP1* function in *Arabidopsis*. Specifically, it finally establishes an off-target candidate locus, responsible for *abp1* mutant phenotypes that have been an integral part of a number of published studies.

With respect to the title:

"early *abp1* mutants" sounds a bit ambiguous. The authors might consider "original *abp1* mutants" instead

I have read this submission. I believe that I have an appropriate level of expertise to confirm that it is of an acceptable scientific standard.

# Peer review: Referee response for version 1

**DOI:** 10.5256/f1000research.7695.r10914

**Published:** 2015-11-02

**Authors:** Richard M. Napier

**Affiliations:** 1 School of Life Sciences, University of Warwick, Coventry, UK

This manuscript examines the genotypic basis behind distinctly different phenotypes which have been reported for arabidopsis mutant lines each annotated as *abp1* knock-out/loss-of-function alleles.  The various mutant lines as homozygotes have been reported over time both as conferring embryo lethality and as displaying no obvious phenotype. Naturally, each outcome translates into very different interpretations of the function and relevance of ABP1. Consequently, clarification of the genotypes behind each phenotype is welcome. This paper maps the mutation sites in each allele and ties embryo lethality to disruption of the adjacent gene *BSM*.  Thus, ABP1 is not necessary for embryo development in arabidopsis.  I applaud the clarity of the conclusion and the suggestion to rename the misleading lines. 

The title is appropriate and factual. The abstract is appropriate. The experimental strategy is direct and sound, methods are described well and suitable statistical analyses have been employed. All the data are presented clearly and are accessible. The text is clear and well structured.

Following the conclusion drawn from the data presented here, there is a discussion on how this and the report of Gao *et al *affect our understanding of the biological role of ABP1 *in planta*. It is clear that in the case of ABP1 molecular genetic techniques have provided misleading genotypes. Thank goodness this is now cleared up. The case made in the discussion is that these specific false leads do not undermine all ABP1 results where the results are based on different genotypes and complementary tools. This is in stark contrast to the case suggested by the somewhat provocative title of the Gao paper. The phenotypes considered differ in timing and detail, but this paper and that of Gao *et al *are linked by revelations clarifying *ABP1* mutant genotypes. The contrasting perspective offered by the discussion contributes to a healthy, objective dialogue. 

Minor points:

Throughout I believe ‘Normanski’ should read Nomarski optics. 

In the results section, text associated with the 35S inserts from SASKATOON accessions should refer to Fig 2A (not 2B).

Technical replicates are used for the qPCR data, and where biological replicates are shown there is clearly more variation than between technical replicates, but interpretations of the result are not affected by this level of uncertainty.  However, I am not sure the use of “Surprisingly” is necessary in the text (page 5, right column) considering the qPCR data for BSM transcripts in Fig 2B.  “In contrast to the loss of ABP1 transcript…” might be more appropriate at this stage of the narrative.  Perhaps reordering consideration of the two sets of data (especially given the ABP1 data are shown first in the figure) would help here.

I have read this submission. I believe that I have an appropriate level of expertise to confirm that it is of an acceptable scientific standard.

# Peer review: Referee response for version 1

**DOI:** 10.5256/f1000research.7695.r10911

**Published:** 2015-11-02

**Authors:** Lars Ostergaard

**Affiliations:** 1 Department of Crop Genetics, John Innes Centre, Norwich, UK

This article provides a clear introduction to the current controversy regarding the Auxin Binding Protein1 (ABP1). The authors have carried out a thorough genetic analysis which shows that the embryo lethality of the *abp1-1* and *abp1-1s* mutants previously assigned to the loss of ABP1 function is in fact due to loss of the neighbouring gene, *BELAYA SMERT* ( *BSM*).

I have read this submission. I believe that I have an appropriate level of expertise to confirm that it is of an acceptable scientific standard.
